# Near-infrared fluorescence image-guidance in anastomotic colorectal cancer surgery and its relation to serum markers of anastomotic leakage: a clinical pilot study

**DOI:** 10.1007/s00464-019-06673-6

**Published:** 2019-02-01

**Authors:** Jacqueline van den Bos, Audrey C. H. M. Jongen, Jarno Melenhorst, Stéphanie O. Breukink, Kaatje Lenaerts, Rutger M. Schols, Nicole D. Bouvy, Laurents P. S. Stassen

**Affiliations:** 1 Department of Surgery, Maastricht University Medical Center, P. Debyelaan 25, 6229 HX Maastricht, The Netherlands; 2 NUTRIM School for Nutrition, Toxicology and Metabolism, Maastricht University, Maastricht, The Netherlands; 3 Department of Plastic, Reconstructive and Hand Surgery, Maastricht University Medical Center, Maastricht, The Netherlands

**Keywords:** Near-infrared fluorescence, NIRF, Colorectal cancer surgery, Anastomotic leakage, CRP, I-FABP, Calprotectin

## Abstract

**Objective:**

Near-infrared fluorescence (NIRF) imaging using indocyanine green (ICG) might help reduce anastomotic leakage (AL) after colorectal surgery. This pilot study aims to analyze whether a relation exists between measured fluorescence intensity (FI) and postoperative inflammatory markers of AL, C-reactive protein (CRP), Intestinal fatty-acid binding protein (I-FABP), and calprotectin, to AL, in order to evaluate the potential of FI to objectively predict AL.

**Methods:**

Patients scheduled for anastomotic colorectal cancer surgery were eligible for inclusion in this prospective pilot study. During surgery, at three time points (after bowel devascularization; before actual transection; after completion of anastomosis) a bolus of 0.2 mg/kg ICG was administered intravenously for assessment of bowel perfusion. FI was scored in scale from 1 to 5 based on the operating surgeon’s judgment (1 = no fluorescence visible, 5 = maximum fluorescent signal). The complete surgical procedure was digitally recorded. These recordings were used to measure FI postoperatively using OsiriX imaging software. Serum CRP, I-FABP, and calprotectin values were determined before surgery and on day 1, 3, and 5 postoperative; furthermore, the occurrence of AL was recorded.

**Results:**

Thirty patients (*n* = 19 males; mean age 67 years; mean BMI 27.2) undergoing either laparoscopic or robotic anastomotic colorectal surgery were included. Indication for surgery was rectal—(*n* = 10), rectosigmoid—(*n* = 2), sigmoid—(*n* = 10), or more proximal colon carcinomas (*n* = 8). Five patients (16.7%) developed AL (*n* = 2 (6.6%) grade C according to the definition of the International Study group of Rectal Cancer). In patients with AL, the maximum fluorescence score was given less often (*P* = 0.02) and a lower FI compared to background FI was measured at 1st assessment (*P* = 0.039). However, no relation between FI and postoperative inflammatory parameters could be found.

**Conclusion:**

Both subjective and measured FI seem to be related to AL. In this study, no relation between FI and inflammatory serum markers could yet be found.

Anastomotic leakage (AL) is the most feared complication in colorectal (cancer) surgery. The severity of leakage may vary from a contained peri-anastomotic fluid collection to generalized peritonitis. Furthermore, long-term morbidity including stricture formation, bowel dysfunction, and an increased chance of locoregional cancer recurrence has been described [1–3].

Clinically apparent anastomotic insufficiency occurs in 5.6–11.9% of all colorectal resections [4–6]. As reported in literature, up to 32% of patients with AL die because of this postoperative complication [4]. These numbers show the urgent need to prevent AL. Prevention starts with eliminating provoking factors. Decreased blood perfusion at the anastomosis is such a factor that contributes to anastomotic leakage [7–9]. Therefore, it is hypothesized that a better visualization of the vascularization of the anastomosis will aid in preventing anastomotic leaks.

Intraoperatively, the selection of an optimal site for anastomosis with adequate perfusion is now dependent on subjective clinical indicators such as color of the bowel, bleeding of the resection margins, and palpable or visible pulsations of the mesenteric arterial vessels (i.e., the clinical judgement of the surgeon). Karliczek et al. [8] discussed that there is a lack of reliable intraoperative predictive test for AL by the operating surgeon.

Earlier studies used Doppler ultrasound and laser Doppler to assess colorectal anastomotic vascularization [9]. Peri-anastomotic tissue oxygenation has been evaluated, but has not been incorporated in routine practice due to the complexity and limited effectivity of the technique [10]. There is need for a technique that can accurately and consistently assess bowel perfusion at the anastomotic site in real time.

Near-infrared fluorescence (NIRF) imaging allows surgeons to visualize the micro-perfusion of the bowel in real time [11]. This technique, with perioperative indocyanine green (ICG) administration, has been shown feasible in assessing the vascularization of the bowel to be anastomosed. Fluorescence angiography with perioperative ICG administration seems to improve the outcome of laparoscopic anastomotic bowel surgery, in terms of safety and efficiency [11, 12]. However, in studies confirming the lower incidence of AL, no objective quantification of the signal was performed [1, 2, 7, 13–15]. Such quantification is desired to make the technique a reliable tool to accurately and consistently assess bowel perfusion at the anastomotic site in real time.

C-reactive protein (CRP) is an early predictor of postoperative infection and has been shown to correlate with AL [16–18]. Intestinal fatty-acid binding protein (I-FABP) is a marker for enterocyte damage [19, 20]. Earlier research showed a significant higher I-FABP at day 3 postoperatively in patients with AL [21]. In the same study, calprotectin levels were elevated on day 2 up to day 5 in patients with AL.

In this pilot study, we aim to analyze whether a relation exists between the objectively quantified signal of ICG fluorescence angiography during laparoscopic or robotic colorectal surgery and the levels of the serum markers related to anastomotic leakage: CRP, I-FABP, and calprotectin, in order to evaluate the potential of FI to objectively predict AL. To our knowledge, this is the first effort to assess the existence of such a correlation.

## Materials and methods

All patients provided written informed consent. This study was approved by the Medical Ethical Committee of Maastricht University Medical Center and conducted according to the Declaration of Helsinki (October 2013, Fortaleza). Included patients were part of the REVEAL study [22], which is registered at clinicaltrials.gov (NCT02347735).

### Study subjects

Colorectal cancer patients scheduled for either laparoscopic or robotic anastomotic surgery were eligible for inclusion. Patients with inflammatory bowel disease were excluded because this could influence plasma levels of the inflammatory markers. Additional exclusion criteria were known iodine or indocyanine green hypersensitivity, impaired renal function (eGFR < 45), pregnancy or breast-feeding (all are contraindications for the use of ICG), and inability to give informed consent. None of the included patients used non-steroidal anti-inflammatory drugs (NSAIDs) or corticosteroids. Patient characteristics (sex, age, BMI, medical history, tumor location) and surgical characteristics (type of surgery, anastomotic technique, ostomy, operation time, conversion rate) were collected.

### Surgical technique

The surgical procedures were performed by experienced laparoscopic colorectal surgeons and started as usual in white light. In laparoscopic cases, a system equipped with fluorescence imaging (Karl Storz GmbH & CO. KG, Tuttlingen, Germany) with a xenon-based light source (D-light P) was used and in robotic surgeries, the da Vinci® System with Firefly fluorescence imaging (Surgical Intuitive, Sunnyvale, United States of America) was used. Surgery was performed as usual except for three time points, at which a bolus of 0.2 mg/kg ICG (Verdye; Diagnostic Green Aschheim, Germany) was administered. These three set time points were:

1. Directly after transection of all the vessels supplying the bowel segment to be resected (i.e., after devascularization of the segment).
2. Just before actual transection.
3. Directly after the anastomosis is made.

After each gift of ICG, the system was switched to NIRF mode and the surgeon was asked to rate the fluorescence intensity of the bowel both proximal and distal to the planned proximal transection line or stapler line on a scale from 1 to 5. Herein, a score of 1 meant no fluorescent signal, while a score of 5 meant ‘maximum fluorescent signal.’ Also, the surgeon was asked whether he or she changed the location of transection based on the visualized fluorescent signal.

When proximal transection was performed extra-corporally, the operative room was completely darkened in order to capture the fluorescent signal with the respective imaging device.

### Fluorescence intensity measurement

Postoperatively, the images of the bowel were assessed with OsiriX imaging software (Pixmeo, Geneva, Switzerland) to quantify the FI. This software measures the gray value in arbitrary units (AU). At the aforementioned three time points, the FI was measured approximately 1 cm proximal and 1 cm distal from the proximal transection point or anastomosis and of the background (abdominal wall or fat directly surrounding the relevant bowel part) in a screenshot taken approximately 1 min after ICG administration. In the right hemicolectomies, the distal transection point was used. In case of extracorporeal proximal transection, also three points in the direct surrounding of the bowel were measured as background. The target-to-background ratio (TBR) was defined as the mean FI in arbitrary units (A.U.) of the bowel wall 1 cm proximal or distal from the planned proximal (or in right hemicolectomies distal) transection location or 1 cm from the staple line of the anastomosis minus the FI of the background divided by the mean FI in the background (abdominal wall or fat directly surrounding the relevant bowel part).

### Definition anastomotic leakage

The patients were followed up to 30 days after surgery, during which the occurrence of complications and especially AL was recorded. Clinically relevant AL was defined and classified according to the definition from the International Study group of Rectal Cancer [23]. In this definition, AL is a defect of the anastomotic wall integrity at the colorectal or colo-anal anastomotic site leading to a communication between the intra-and extraluminal compartments. A pelvic abscess close to the anastomosis is also considered AL. We applied this definition also to the included colocolic and ileocolic anastomoses in our study. A distinction is made between the severity of AL based on the intervention required:

- Grade A: Anastomotic leakage requiring no active therapeutic intervention;
- Grade B: Anastomotic leakage requiring active therapeutic intervention but manageable without relaparotomy;
- Grade C: Anastomotic leakage requiring relaparotomy [23].

### Analysis of inflammatory markers

Venous blood was drawn at hospital admission (preoperative sample) and on day 1, 3, and 5 after surgery, unless the patient was discharged home earlier. C-reactive protein (CRP) plasma levels were determined by using Immunoturbidimetric assay for the in vitro quantitative determination of CRP in human serum and plasma (Roche diagnostics, Germany). Serum calprotectin concentration was determined using a commercially available calprotectin ELISA (Hycult Biotechnology, Uden, The Netherlands). Intestinal fatty-acid binding protein (I-FABP) plasma levels were determined using an in-house ELISA that selectively detects human I-FABP [21].

### Statistical analysis

Basic descriptive statistics were applied to calculate means and standard deviation of the baseline characteristics. Because of the small sample size, non-parametric tests were used to analyze the data retrieved in this study. To compare continuous variables between two groups, the Mann–Whitney test was performed. To compare categorical outcomes between two groups, a Pearson Chi-square crosstab was used. The relation between the measured fluorescence intensity and the values of the inflammatory markers was evaluated using a regression analysis. *P* < 0.05 was considered to denote statistical significance. All statistical analyses were performed with SPSS version 23 (IBM, Armonk, NY, USA).

## Results

### Study population

Thirty patients were included with a mean age of 67.2 (SD 8.2) years of whom 19 (63%) were male. All underwent elective surgery for colorectal cancer, of which 10 (33.3%) concerned sigmoid carcinoma, 10 (33.3%) rectum carcinoma, 5 (16.7%) colon carcinoma in the right hemi-colon, 3 (10%) cecum carcinoma, and 2 (6.7%) rectosigmoid carcinoma. Five patients were treated with concomitant chemoradiotherapy before surgery, 4 with only chemotherapy (CAPOX–bevacizumab), and 3 received only radiotherapy (5 × 5 Gy). A conversion to open surgery was needed in 7 patients in whom a laparoscopic procedure was started, due to unclear anatomy in obese patients. Further baseline characteristics and surgical characteristics are summarized in Table 1.

**Table 1:** Baseline characteristics and surgical characteristics

	n (%)	Mean (SD)
Male	19 (63.3)	
Age, years		67.2 (8.2)
Weight, kg		82 (16.4)
BMI, kg/m^2^		27.2 (4.9)
Smoking	12 (40)	
Both preoperative chemo- and radiotherapy	5 (16.6)	
Preoperative chemotherapy	4 (13)	
Preoperative radiotherapy	3 (10)	
Location of suspected tumor		
Cecum	3 (10)	
Right hemi-colon	5 (16.7)	
Sigmoid	10 (33.3)	
Rectosigmoid	2 (6.7)	
Rectum	10 (33.3)	
Type of surgery		
Laparoscopic right hemicolectomy	8 (26.7)	
Laparoscopic sigmoid resection	11 (36.7)	
Laparoscopic low anterior resection	4 (13.3)	
Robot-assisted low anterior resection	7 (23.3)	
Deviating stoma	11 (36.7)	
Type of anastomosis		
Side-to-end	16 (53.3)	
End-to-end	9 (30)	
Side-to-side	5 (16.7)	
Conversion to open surgery	7 (23.3)	

### Near-infrared fluorescence imaging

A bolus of 0.2 mg/kg ICG was administered intravenously. Thus, the total dose ranged between 10.3 and 21.8 mg with a mean of 16.4 mg (SD 3.3). NIRF imaging was aimed to be performed at three time points. In 19 patients, fluorescence imaging was achievable at all three time points. In 10 patients, the first time point was very close to the second time point; therefore in these patients only just before transection (second time point) and after completion of the anastomosis, the bowel was assessed using NIRF imaging, in order to prevent potential accumulation of the dye at the second time point. In two patients, the anastomosis was too low to visualize the NIRF signal. The time between the first and second time point was on average 68.6 min (range 9–201) and between the second and third time point on average 40 min (range 11–84).

### Intraoperative decision making based on fluorescence imaging

In six patients, the dissection location was changed, based on the fluorescent signal. One of these patients developed AL; the remaining 5 patients did not.

### Anastomotic leakage (AL)

In 5 patients (16%), AL occurred. In 2 of these cases, only a small abscess around the anastomosis was seen on CT scan and could be treated non-surgically with intravenous administration of antibiotics and CT-guided drainage. These leakages were considered as grade B AL. In 1 patient, AL could be treated with an endo-sponge applied via endoscopy and was also considered as Grade B AL. The remaining 2 patients (6% of the total study population) needed relaparotomy for AL treatment (grade C). All patients with AL underwent a low anterior resection of a rectum or rectosigmoid carcinoma. Two of the AL patients had undergone preoperative chemoradiotherapy: one preoperative radiotherapy and one preoperative chemotherapy. The remaining patient was not preoperatively treated with neoadjuvant radiotherapy or chemotherapy. There were no statistical differences in age at the time of surgery, preoperative chemotherapy, BMI, administered dose of ICG per bolus (mg), conversion to open surgery, type of anastomosis, TNM classification after surgery, and difficulty of surgery between the patients who developed AL and the patients who did not. There were significantly more smokers in the AL group (*P* = 0.046). The postoperative hospital stay was on average 16 days (median 12 days) in the patients with AL, which was significantly longer compared to the patients without this complication, who stayed on average 5 days (*P* = 0.004). A deviating ileostomy had been constructed more often in the AL group. See Table 2 for an overview of these characteristics.

**Table 2:** Characteristics of patients with anastomotic leakage compared to those without

	No AL (total 25)	AL (total 5)	*P* value
Male n	15 /25	4 /5	0.397
Age, years (median (IQR))	65.5 (11.5)	65.0 (17)	0.787
BMI, kg/m^2^ (median (IQR))	28.3 (5.27)	24.4 (14.1)	0.627
Smoking n	8 /25	4 /5	0.046*
Preoperative chemoradiation	3/25	2/5	0.183
Preoperative chemotherapy	3 /25	1/5	0.538
Preoperative radiotherapy	2/25	1/5	0.433
Indication for surgery			0.058
Cecum carcinoma	3	0	
Right hemi-colon carcinoma	5	0	
Sigmoid carcinoma	10	0	
Rectosigmoid carcinoma	1	1	
Rectum carcinoma	6	4	
Type of surgery			0.107
Laparoscopic right colectomy	8	0	
Laparoscopic sigmoid resection	10	1	
Laparoscopic low anterior resection	2	2	
Robotic low anterior resection	5	2	
Change of dissection place	5 /25	1 /5	1.000
Ileostomy	7/25	4/5	0.028*
Type of anastomosis			
Side-to-end	13	3	
Side-to-side	9	0	
End-to-end	3	2	
Conversion to open surgery	6 /25	1 /5	0.847
Surgical time, minutes ((median (IQR))	203.5 (95)	287.0 (358)	0.344
Postoperative hospital stay, days (median (IQR))	4 (2.25)	12 (22)	0.004*

*Statistical significant difference (*P* < 0.05)

Other postoperative complications were postoperative ileus in one patient, which resolved without further consequence. The two patients who needed relaparotomy due to anastomotic leakage both developed pneumonia after the second surgery which was treated with antibiotics.

### Subjective fluorescence intensity (FI)

The FI was scored by the surgeon on a scale from 1 to 5. In all cases, in all three assessments, this score ranged between 3 and 5 for the bowel proximal to the proximal transection site, or distal transection site in right hemicolectomies. In the second assessment, the subjective score given for the FI was 4 in four of the anastomotic leakage cases. In one of the anastomotic leakage cases, a score of 5 was given, this was one of the cases in which only an abscess close to the anastomosis was present. In the patients who did not develop AL the maximum score of 5 was given in 16 cases (73%), which was significantly more often compared to the AL group (*P* = 0.02).

In both the first and third assessment, no significant differences in subjective fluorescence intensity between the patients with and without anastomotic leakage were seen. See Table 3 for the given scores.

**Table 3:** Subjective fluorescence intensity (FI) scores and anastomotic leakage (AL)

	FI score 3	FI score 4	FI score 5	Total no of patients assessed
1st assessment				
No AL	1	5	10	16
AL	0	1	3	4
2nd assessment				
No AL	3	4	18	25
AL	0	4	1	5
3rd assessment				
No AL	2	11	11	24
AL	1	1	2	4

The fluorescence intensity on first and second assessment is scored on the distal side of the distal anastomosis in laparoscopic right hemicolectomies, while on the proximal site of the proximal anastomosis in all remaining procedures

### Measured fluorescence intensity

Next to subjective scoring by the surgeon, the FI of the bowel proximal and distal from the proximal transection or distal in right hemicolectomies and the background was measured by image quantification. As shown in Tables 4, 5, 6, and 7, the FI in patients without AL was higher at almost all measurements compared to the FI in patients who develop AL. This difference is however only statistically significant when we take the background into account and assess the bowel distal from the proximal anastomosis in the 1st assessment (*P* = 0.039) (see Table 7).

**Table 4:** Fluorescence intensity (FI) bowel proximal from the proximal transection line and anastomotic leakage (AL) (distal from distal transection line in right hemicolectomies, thus side from the transection line that stays in the patient)

	Median FI no AL	Median FI AL	*P* value
1st assessment	38.8 (61.2)	34.2 (28.7)	0.494
2nd assessment	49.2 (45.0)	44.1 (94.6)	0.385
3rd assessment	86.5 (54.8)	99.5 (68.1)	0.336

Median fluorescence intensity (interquartile range)

**Table 5:** Fluorescence intensity (FI) bowel distal from the proximal transection line and anastomotic leakage (AL) (proximal from the distal transection line in right hemicolectomies, thus side from the transection line that will be resected)

	Median FI no AL	Median FI AL	*P* value
1st assessment	31.7 (23.7)	36.7 (27.1)	0.064
2nd assessment	40.8 (32.4)	64.5 (72.8)	0.448
3rd assessment	59.9 (37.6)	55.9 (30.5)	0.952

Median fluorescence intensity (interquartile range)

**Table 6:** Target-to-background ratio (TBR) bowel proximal from the proximal transection line and anastomotic leakage (AL) (distal from distal transection line in right hemicolectomies, thus side from the transection line that stays in the patient)

	Median TBR no AL	Median TBR AL	*P* value
1st assessment	2.8 (4.7)	1.1 (1.4)	0.820
2nd assessment	2.2 (13)	0.5 (2.1)	0.229
3rd assessment	1.4 (2.6)	1.4 (1.0)	0.669

Median fluorescence intensity (Interquartile range)

**Table 7:** Target-to-background ratio (TBR) bowel distal from the proximal transection line and anastomotic leakage (AL) (proximal from distal transection line in right hemicolectomies, thus the side from the transection line that will be resected)

	Median TBR no AL	Median TBR AL	*P* value
1st assessment	3.4 (6.9)	1.2 (1.3)	0.039*
2nd assessment	2.6 (13.5)	1.8 (4.0)	0.482
3rd assessment	1.4 (2.9)	0.9 (0.1)	0.364

Median fluorescence intensity (interquartile range)

*Statistical significant difference (*P* < 0.05)

### Inflammatory serum markers

As shown in Table 8, CRP was significantly higher on postoperative day 5 in anastomotic leakage patients. As is shown in Table 8, all other measured markers do not show a statistical difference between the occurrence of anastomotic leakage and serum levels on day 1, 3, or 5 (Table 8).

**Table 8:** Serum markers anastomotic leakage versus no anastomotic leakage

	No anastomotic leakage	Anastomotic leakage	*P* value
Mean CRP			
Preoperative (mg/ml) ± SD	2.9 ± 2.3	2.1 ± 0.9	0.69
Postoperative day 1 (mg/ml) ± SD	87.4 ± 35.8	87.4 ± 35.3	0.90
Postoperative day 3 (mg/ml) ± SD	101.1 ± 48.0	183.6 ± 123.5	0.09
Postoperative day 5 (mg/ml) ± SD	48.5 ± 24.66	152.8 ± 90.7	0.01*
Mean calprotectin			
Preoperative (ng/ml (SD)	822.9 ± 765.4	3451.6 ± 3987.1	0.41
Postoperative day 1 (ng/ml) ± SD	7587.3 ± 17589.9	7743.5 ± 12026.8	1.0
Postoperative day 3 (ng/ml) ± SD	2467.0 ± 1430.3	1683.3 ± 1549.4	0.15
Postoperative day 5 (ng/ml) ± SD	3326.7 ± 2783.0	2756.1 ± 1974.3	0.68
I-FABP			
Preoperative (pg/ml) ± SD	805 ± 376.6	520.1 ± 516.1	0.16
Postoperative day 1 (pg/ml) ± SD	644.7 ± 370.4	383.2 ± 160.4	0.14
Postoperative day 3 (pg/ml) ± SD	574.8 ± 431.4	373.9 ± 335.3	0.31
Postoperative day 5 (pg/ml) ± SD	496.9 ± 291.4	143.0 ± 49.6	0.05

*Statistical significant difference (*P* < 0.05)

### Fluorescence intensity in relation to inflammatory serum markers

The relation between the respective serum markers CRP, Calprotectin and I-FABP, and the fluorescent signal was assessed using a regression analysis. For none of the serum markers on any time point, a significant relation could be found with the fluorescence both proximal and distal or measured as TBR, with one exception. At the third time point, after the anastomosis is made, a higher TBR of the distal part of the anastomosis was related to a lower serum calprotectin level on postoperative day 5 (*P* = 0.046).

## Discussion

NIRF angiography with ICG is used more and more in colorectal surgery as this technique holds the potential to lower the risk for anastomotic leakage [11]. However, it is unknown when the bowel is fluorescent enough to actually predict optimal anastomotic healing. In this respect, a cut-off value is desirable. To come to such consensus, we first need to know whether a “more fluorescent bowel” actually is indicative for a lower risk for anastomotic leakage and accompanying lower postoperative inflammatory parameters. Therefore, we performed this clinical pilot study, in which we aimed to correlate the signal strength of fluorescence angiography with the occurrence of AL and with the level of the serum markers that are thought to be predictive of AL: CRP, calprotectin, and I-FABP. The fluorescence intensity was measured after administration of 0.02 mg/kg ICG intravenously at three time points. CRP, calprotectin and I-FABP were determined preoperatively and on day 1, 3, and 5 after surgery. Follow-up for anastomotic leakage was until 30 days postoperatively.

In the current study, 5 (16.7%) patients developed AL. Three of these leakages were considered a grade B leakage, while the remaining two were grade C anastomotic leakages which needed relaparoscopy. The number of patients with AL is somewhat higher compared to the literature (5.6–11.9%) [4–6], and especially when compared to other studies in which NIRF angiography is used during colorectal surgery [11]. This could be explained by the fact that both grade B and grade C anastomotic leakage were taken into account in this study, while other studies may have only considered grade C leakage only. In addition, 40% of patients smoked, which is a risk factor for developing AL [4, 24]. Remarkably, preoperative radiotherapy, which is also a known risk for leakage was not identified as such in our study. The limited number of patients may have contributed to this discrepancy.

In all cases, the subjective score for FI proximal from the (planned) proximal transection line or distal from distal transection line (in right hemicolectomies), ranged between 3 and 5 on a scale from 1 to 5. For the bowel proximal from the transection line, the maximum score of 5 was given significantly more often in the patients that healed without a leak. This is in line with the study by Ris et al. [2] in which the fluorescence score ranges from ‘good,’ ‘average,’ to ‘bad.’ In all patients, the fluorescence was scored ‘good’ by the surgeon and none of the patients developed AL. Other studies scored the fluorescence intensity as ‘good enough’ or ‘not good enough’ based on subjective score of the surgeon. In these studies, if the fluorescence intensity was considered not good enough, the dissection place was changed [13, 25–29]. The anastomotic leakage rate in patients with changed dissection level was equal [13, 25–27, 29] or higher [28] compared to the patients in which no change was needed after NIRF imaging. A possible explanation for a higher leak rate after change of transection location may be the fact that a clearly insufficiently perfused bowel end may be indicator of a longer segment of moderate perfusion. This emphasizes the need for cut-off values for level of perfusion [11]. In the current study, revision of surgical plan based on the FI of the bowel occurred in six patients. One of these developed AL. These numbers are too low to draw conclusions.

In the present study, the objectively measured FI was higher in the patients who did not develop AL. This difference was only significant when taking the background into account and expressing data as TBR. By using TBR, a correction is made for the distance between the endoscope and the bowel. A shorter distance between the laparoscope and the target causes a higher FI [30]. By taking the background into account, this effect is minimized, since the background will also show increased fluorescence intensity when holding the laparoscope closer to the tissue. Such correction of the signal seems necessary, as in practice, it is not always possible to exactly measure and standardize the distance to the bowel.

According to literature, the CRP trajectory is highly predictive for AL [6]. In the current series, CRP on postoperative day 5 was significantly higher in patient who developed AL. This observation is in line with findings reported in literature. An earlier study by Reisinger et al. [21] showed a rise in postoperative CRP in patients with an anastomotic leak, which was only significant on day 4. Bigin et al. [31] showed a significant higher CRP on postoperative day 3, but unfortunately CRP was not measured on postoperative day 5. Reynolds et al. [17] found a significantly higher CRP level in patients with grade C AL on day 5 up to day 7. Unfortunately, in the current study, no relation of CRP and subjective or measured fluorescence intensity or TBR could be found.

Calprotectin is a non-specific marker for acute inflammation [32]. An earlier study by Reisinger et al. [21] showed higher calprotectin levels on postoperative day 2, 3, 4, and 5 in patients who developed AL. Similar results were found by Cikot et al. [33]. In the current study however, no statistical difference in serum calprotectin levels could be found between patients with and without anastomotic leakage on postoperative day 1, 3, or 5. Interestingly, serum calprotectin levels on postoperative day 5 were negatively correlated with TBR of the distal part of the anastomosis. This relation needs to be confirmed in a larger study to determine its clinical relevance.

I-FABP is an intestinal cell damage marker [21]. An earlier study by Reisinger et al. [21] showed significantly higher I-FABP levels preoperatively in patients who developed anastomotic leakage. The I-FABP levels remained higher in these patients after surgery, but only statistically significant on postoperative day 3. In the current study however, I-FABP levels were lower at all time points in the patients who developed anastomotic leakage, although this difference was not significant. Moreover, we could not detect a significant relation between FI and serum I-FABP values in this study.

A possible explanation for this discrepancy might be the low specificity of I-FABP. This marker for enterocyte damage is not only elevated in case of inflammation, but also in patients with colorectal cancer compared to patients without colorectal cancer [34]. When only taking the patients with a grade C anastomotic leakage into account, a (non-statistically significant) higher I-FABP value is found in the leakage patients. More research is needed to determine its place in the management of the colorectal operative patient.

A higher fluorescence intensity was found in all cases in the bowel at the side that will remain in the patients after surgery. This means that the devascularized bowel results in a lower signal, indicating that the fluorescent signal does reflect bowel perfusion.

This study has some limitations. First, it was designed as a pilot study to explore whether there is a potential relation between FI and inflammatory serum markers that correspond with AL. A relatively small and heterogenous group of patients was included. With the markers and the patient group chosen here, it was not yet possible to identify a cut-off value of fluorescent signal that indicates an optimal anastomotic healing potential. A more homogenous patient group (e.g., only right hemicolectomies or rectal resections) might have given different results. Another factor of influence could be that only the measurements at single time points were performed, and not an evaluation of the signal intensity over time after administration of the respective boluses of ICG. The small number of included patients can be an explanation for finding few statistical significant differences for most outcome parameters.

In this study, several time points of ICG administration were chosen. Although arterially the dye washes out very quickly—which is evident when observed with the naked eye—it cannot be ruled out that some retention of the dye occurred. Although this phenomenon will take place in both the bowel wall and the background, this may have influenced the objective measurements. The time between the first and second dose administration was on average 68.6 min (range 9–201) and between the second and third bolus of ICG 40 min (range 11–84). A cut-off value for the optimal interval is not known in literature.

In conclusion, both subjective and measured FI seem to be related to the occurrence of AL. In this pilot study, no relation between FI and inflammatory serum markers could yet be found nor a cut-off value of FI predicting AL. A larger study based on the current study, with a single time point of measurement in a homogenous population seems preferable to enhance the chance that an objective cut-off value of the fluorescent signal to predict AL will be identified. Ideally, future research identifies such cut-off value, but also leads to presenting the objective result of fluorescence measurement real time to the surgeon, thereby supplying the surgeon with essential per-operative information for the optimal transection point to prevent anastomotic leakage.

## Abbreviations

NIRF: Near-infrared fluorescence imaging
ICG: Indocyanine green
CRP: C-reactive protein
I-FABP: Intestinal fatty-acid binding protein
AL: Anastomotic leakage
